# The drug likeness analysis of anti-inflammatory clerodane diterpenoids

**DOI:** 10.1186/s13020-020-00407-w

**Published:** 2020-12-09

**Authors:** Zheling Feng, Jun Cao, Qingwen Zhang, Ligen Lin

**Affiliations:** State Key Laboratory of Quality Research in Chinese Medicine, Institute of Chinese Medical Sciences, University of Macau, Avenida da Universidade, Taipa, Macau, 999078 People’s Republic of China

**Keywords:** Clerodane diterpenoids, Anti-inflammation, Drug-likeness, SwissADME

## Abstract

Inflammation is an active defense response of the body against external stimuli. Long term low-grade inflammation has been considered as a deteriorated factor for aging, cancer, neurodegeneration and metabolic disorders. The clinically used glucocorticoids and non-steroidal anti-inflammatory drugs are not suitable for chronic inflammation. Therefore, it’s urgent to discover and develop new effective and safe drugs to attenuate inflammation. Clerodane diterpenoids, a class of bicyclic diterpenoids, are widely distributed in plants of the Labiatae, Euphorbiaceae and Verbenaceae families, as well as fungi, bacteria, and marine sponges. Dozens of anti-inflammatory clerodane diterpenoids have been identified on different assays, both in vitro and in vivo. In the current review, the up-to-date research progresses of anti-inflammatory clerodane diterpenoids were summarized, and their druglikeness was analyzed, which provided the possibility for further development of anti-inflammatory drugs.

## Background

Inflammatory diseases include a vast array of disorders and defense reaction of organisms against external stimulations that are characterized by inflammation symptoms, such as allergy, autoimmune diseases, asthma, glomerulonephritis, hepatitis, inflammatory bowel disease (IBD), reperfusion injury and transplant rejection [1–3]. Long term low-grade inflammation has been considered to play a deteriorated role in many diseases, such as aging, cancer, metabolic disorders, and neurodegeneration [4–7]. The occurrence of chronic diseases has triggered prolonged inflammation that induces the expression of robust pro-inflammatory mediators and cytokines [8, 9], which lead to the pathogenesis of inflammation-associated chronic diseases. Tumor necrosis factor (TNF)-α is one of the most potent pro-inflammatory cytokines and signals [10, 11]. Through binding to its receptors, TNFR1 and TNFR2, TNF-α plays critical roles in apoptosis, cell proliferation and immune responses [11, 12]. Interleukin-1β (IL-1β) is one of the inflammatory markers belonging to the IL-1 family of cytokines [13, 14]. IL-6 and IL-12 display a pro-inflammatory action via stimulating IL-1 secretion [15]. IL-10, as the most important anti-inflammatory cytokine, represses pro-inflammatory responses and limits inflammation-induced tissue disruptions [16, 17]. Prostaglandin E2 (PGE2) is derived from arachidonic acid produced by cyclooxygenase (COX)-1 and/or COX-2 [18], which is a principal mediator of inflammation in diseases such as rheumatoid arthritis and osteoarthritis [19]. Nitric oxide (NO) is free radical acting as a cellular signaling molecule in inflammation process [20, 21]. NO is synthesized from I-arginine through the action of nitric oxide synthase (NOS) family [22], which has been associated with the pathogenesis and progression of inflammatory-related diseases [20, 23]. Macrophages exit throughout the body, which play important roles in tissue development, inflammation and anti-pathogenic defense [24–26]. A variety of biologically active molecules related to the beneficial and harmful consequences of inflammation are produced by macrophages. Therefore, therapeutic intervention for macrophages and their related products has attracted widespread attention in the treatment of inflammatory diseases. [24, 27].

**Fig. 1:**
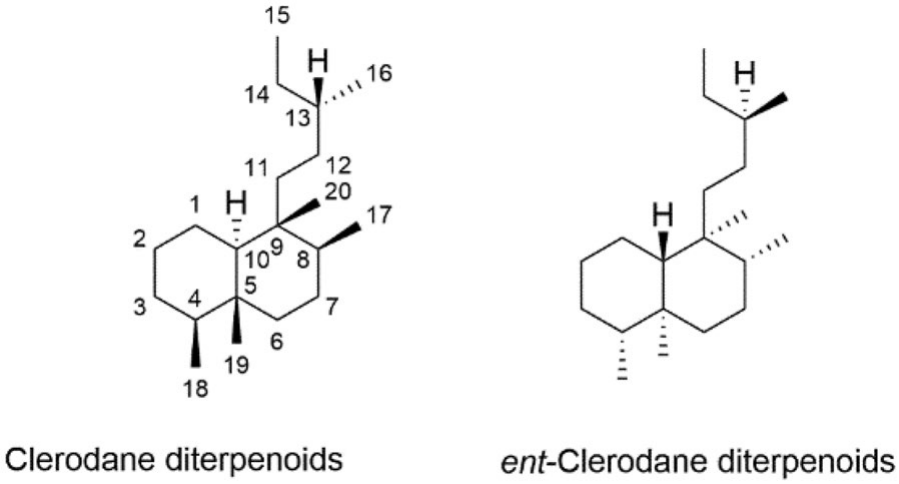
Skeletons of clerodane diterpenoids and *ent*-diterpenoids

Currently, the clinically used inflammation-treating drugs include non-steroidal anti-inflammatory drugs (NSAID) and glucocorticoids (SAID). Both classes of anti-inflammatory drugs could induce unpleasant side effects, and not suitable for chronic use [28–30]. Thus, it’s urgent to discover and develop new effective and safe drugs to alleviate inflammation. Tons of scientific evidence has indicated that natural medicines represent a big treasure to develop potential therapeutic agents for inflammation-related diseases [31, 32]. The clerodane diterpenoids are a large class of naturally occurring bicyclic diterpenoids (Fig. 1), found in lots of plant species, especially the families of Labiatae, Euphorbiaceae and Verbenaceae [33]. More and more research interests have been attracted in recent years for their wide and potent biological activities [34]. Salvinorin A, a clerodane diterpenoid isolated from *Salvia divinorum*, was found as an agonist of the κ-opioid receptor. It is the first opioid receptor agonist without nitrogen and non-alkaloid hallucinogen. Many clinical trials results support the physiological effects of salvinorin A is mediated through κ-opioid receptor‒serotonin-2A pathway [35–40]. A lot of studies have disclosed the potential anti-inflammatory activity of clerodane diterpenoids. Herein, an up-to-date and comprehensive review of clerodane diterpenoids with anti-inflammatory property was provided, and their drug-likeness was analyzed through SwissADME, which enable further development of clerodane diterpenoids for the treatment of inflammation related diseases.

## Clerodane diterpenoids with anti-inflammatory property

**Fig. 2:**
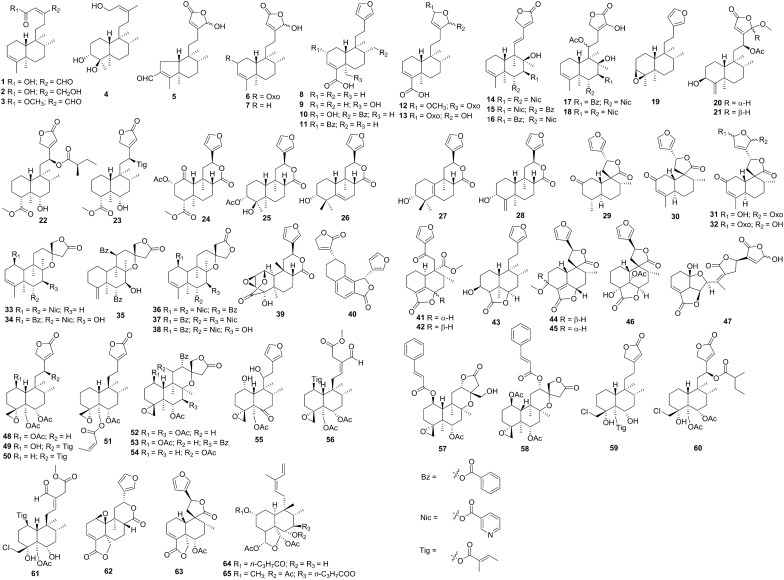
Structures of clerodane diterpenoids with anti-inflammatory activity

Data was collected from Web of Science, Google Scholar, Scopus, and Pubmed via using the keywords clerodane diterpenoid and inflammation, and a total of 65 clerodane diterpenoids were found with anti-inflammatory property (Fig. 2). Either in vitro or in vivo bioassays have been used to determine the anti-inflammatory activity of clerodane diterpenoids. To better organize the review, the clerodane diterpenoids were introduced based on the anti-inflammatory bioassays (Table 1).

**Table 1:** Clerodane diterpenoids with anti-inflammatory activity

No	Model/Method	Dose	Outcomes	Mechanisms	Ref
**14**	LPS-induced murine microglial BV-2 cells	30.9 μM	Inhibited NO production		[44]
**16**		31.2 μM			
**17**		30.3 μM			
**18**		27.5 μM			
**38**		34.7 μM			
**48**		45.5 ± 3.6 μM			[47]
**49**		34.0 ± 3.2 μM			
**51**		48.5 μM			[45]
**52**		36.0 ± 0.8 μM		Directly interacted and suppressed iNOS	[43]
**53**		30 μM			
**54**		27.3 ± 1.3 μM			
**57**		11.1 ± 0.8 μM			
**58**		30 μM			
**60**		25.3 μM			[46]
**61**		27.2 μM			[45]
**22**		25.8 ± 1.7 μM		Directly interacted and suppressed iNOS and COX2	[47]
**50**		27.0 ± 0.9 μM			
**59**		20.2 ± 2.0 μM			
**1**	LPS-induced RAW264.7 macrophages	31.4 μM	Inhibited NO production		[57]
**4**		25.49 μM			[58]
**7**		31.4 μM			[57]
**14**		26.3 μM			[53]
**15**		27.7 μM			
**16**		22.8 μM			
**19**		32.19 μM			[54]
**20**		22.92 μM			[50]
**21**		13.25 μM			
**25**		46.43 μM			[54]
**26**		31.99 μM			
**27**		48.85 μM			
**28**		42.04 μM			
**33**		35.6 μM			[53]
**34**		34.2 μM			
**35**		20.6 μM			
**36**		21.5 μM			
**37**		20.2 μM			
**41**		1.2 μM			[51]
**42**		1.6 μM			
**44**		0.82 μM			[91]
**45**		0.54 μM			
**46**		21.9 μM			[52]
**47**		45.1 μM			[59]
**55**		39.3 μM			[56]
**56**		36.2 μM			[55]
**63**		22.4 μM			[52]
**12**		35.35 μM	Inhibited NO, IL-6, IL-1β and TNF-α production in a dose-responsive manner		[50]
**13**		17.49 μM			
**2**	fMLP/CB induced neutrophils	4.40 ± 0.83 μM	Suppressed superoxide generation		[64]
**3**		1.81 ± 0.27 μM			
**5**		13.00 ± 1.77 μM			
**6**		23.95 ± 5.36 μM			
**7**		9.59 ± 3.55 μM			
**8**		4.40 ± 0.56 μM 3.67 ± 0.20 μM	Inhibited superoxide anion generation and elastase release		[65]
**2**		3.06 ± 0.20 μM		Attenuated the phosphorylation of AKT and p38 MAPK	[66]
**9**	TPA-induced CD-1 auricular pavilion mouse ear edema	0.25, 0.5, 1 mg/ear	Reduced edema to 64%		[71]
**10**		0.10 mg/ear	exhibited 70-76% Inhibition of inflammation		[70]
**11**		0.39 mg/ear			
**40**		200 mg/kg	Dose-dependently inhibited ear edema		[73]
**62**		0.50 μg/ear	Inhibited ear edema		[72]
**10**	TPA-induced acute mouse ear edema	2.03 μg/ear	Inhibited IL-1β production, reduced ear thickness, IL-6 and myeloperoxidase accumulation		[81]
**24**	carrageenan-induced paw edema in rats	50–200 mg/kg	Dose-dependently inhibited paw edema.		[73]
**30**		200 mg/kg	Inhibited paw edema and cotton pellet granuloma		[77]
**43**		20 mg/kg	Inhibited inflammation in rat paw		[78]
**64**		0.5, 2.5 mg/kg	Reduced paw edema		[76]
**65**					
**24**	Carrageenan-induced pleurisy in rats	10 mg/kg	Inhibited LTB4 production in exudates and phlogistic process in lung		[79]
**31**	Teleocidin induced mouse ear inflammation	5.6 μg/ear	Inhibited tropical inflammation in mouse ear		[92]
**32**		3.0 μg/ear			
**39**	Indomethacin-induced gastric ulcer in rats	50 mg/kg	Downregulated PGE2, IL-4, IL-10, VEGF, and EGF		[83]
**7**	DSS/OAM induced IBD mice	1.6, 6.4 mg/kg	Reduced iNOS, COX2 expression	Reduced NF-κB gene expression	[85]
**29**	Dextran- or histamine-induced edema	50 mg/kg	Reduced edema		[80]
**24**	A23187-induced LTB4 biosynthesis	1 μM	Dose-dependently inhibited LTB4 biosynthesis		[79]
	Zymosan-induced peritonitis	10 mg/kg	Inhibited myeloperoxidase activity, LTC4 production, cell infiltration, and vascular permeability in the peritoneal cavity, but not the production of PGE2		

Lipopolysaccharide (LPS), a major constituent of the outer leaflet of Gram-negative bacterial outer membrane, is synthesized on the cytoplasmic membrane and transferred to the outer membranes [41, 42]. LPS treated murine microglial BV-2 cells are widely used for anti-inflammatory drug screening and underlying mechanism investigation. Using the above mentioned model, 18 clerodane diterpenoids including scutebarbatine (**14**), scutebarbatine B (**16**), scutebatas B (**17**), scutebarbatine X (**18**), ajugapantin H (**22**), scutebaabaatine W (**38**), ajugapantins E (**48**), ajugapantins F (**49**), ajugapantins G (**50**), ajugacumbin A (**51**), scutelapenes B (**52**), scutelapenes C (**53**), scutelapenes D (**54**), scutelapenes A (**57**), scutelapenes E (**58**), ajugapantin C (**59**), (12*S*,2′′*S*)-6*α*,19-diacetoxy-18-chloro-4α-hydroxy-12-(2-methylbutanoyloxy)-neo-clerod-13-en-15,16-olide (**60**), and 6*α*,19-diacetoxy-4*α*-hydroxy-1*β*-tigloyloxyneo-clerod-12-en-15-oic acid methyl ester-16-aldehyde (**61**) were found to inhibit NO production [43‒46]. Among them, compounds **22**, **50** and **59** were discovered to downregulate iNOS and COX-2 expression [47].

LPS-stimulated murine-derived RAW264.7 macrophages have been widely used as a bioassay to investigate anti-inflammatory activity [48, 49]. Using this model, 29 clerodane diterpenoids including 16-oxocleroda-3,13-dien-15-oic acid (**1**), neoclerod-13*Z*-ene-3*α*,4*β*,15-triol (**4**), 16-hydroxycleroda-3,13-dien-15,16-olide (**7**), 15-methoxypatagonic acid (**12**), 16-hydroxycleroda-3,13-dien-16,15-olide-18-oic acid (**13**), scutebarbatine A (**14**), scutebarbatine Y (**15**), scutebarbatine B (**16**), 3*β*,4*β*:15,16-diepoxy-13(16),14-clerodadiene (**19**), cathayanalactones A (**20**), cathayanalactones B (**21**), crotonolide K (25), furocrotinsulolide A acetate (**26**), 15,16-epoxy-3*β*-hydroxy-5(10),13(16),14-ent-halimatriene-17,(12*S*)-olide (**27**), crotonolide F (**28**), scutebatin C (**33**), scutebarbatine W (**34**), scutebata P (**35**), scutebatin A (**36**), scutebatin B (**37**), 6*S*-crotoeurin C (**41**), crotoeurin C (**42**), 3*S*-methoxylteucbin (**44**), 3*R*-methoxylteucbin (**45**), 19-acetyl-teuspinin (**46**), jamesoniellide Q (**47**), 11-hydroxyfruticolone (**55**), ajugacumbin J (**56**), and 6α-acetoxyteuscordin (**63**) were found to inhibit NO production [50‒59], and compounds **12** and **13** were further found to reduce IL-6, IL-1β, and TNF-α production in dose-responsive manners [50].

Besides being deputed to host defense against microorganisms, neutrophils display fundamental roles both in inflammation and tissue damages [60]. *N*-formylmethionyl-leucyl-phenylalanine (fMLP) is a potent polymorphonuclear leukocyte chemotactic factor and a macrophage activator [61]. Neutrophils function as the first-line defense against invading bacteria fungi and protozoa [62], which are major effector cells to identify novel antibiotics and elucidate the pathogenesis behind neutrophil-mediated inflammatory disorders [63]. In fMLP/CB-stimulated human neutrophils, five clerodane diterpenoids including 16-hydroxycleroda-3,13(14)*E*-dien-15-oic acid (**2**), 16-oxocelroda-3,13(14)*E*-dien-15-oic acid methyl ester (**3**), (4-2)abeo-16-2,13*Z*-clerodadien-15,16-olide-3-al (**5**), 16-3,13*Z*-kolavadien-15,16-olide-2-one (**6**), and 16-hydroxycelroda-3,13-dien-15,16-olide (**7**) from the bark of *Polyalthia longifolia* var. *pendula* showed anti-inflammatory activity [64]. (‒)-Hardwickiic acid (**8**) showed anti-inflammatory activity on neutrophils through suppressing both superoxide anion generation (IC_50_ = 4.40 ± 0.56 μM) and elastase release (IC_50_ = 3.67 ± 0.20 μM) [65]. 16-Hydroxycleroda-3,13(14)*E*-dien-15-oic acid (**2**) was isolated from the same species, which concentration-dependently inhibited the generation of superoxide anion and the release of elastase in fMLP/CB-stimulated human neutrophils, with IC_50_ values of 3.06 ± 0.20 and 3.30 ± 0.48 μM, respectively [66].

TPA (12-*O*-tetradecanoylphorbol 13-acetate) induces keratinocytes proliferation, TNF-α production, and the formation of leukotriene B4 (LTB4), to induce oxidative stress and cutaneous inflammation [67, 68]. Mice overexposed to TPA exhibit an inflammatory phenotype characterized by the increased ear thickness, macrophage infiltration, and epidermal hyperplasia [69]. In TPA-induced CD-1 auricular pavilion mice, two benzoyl ester clerodane diterpenoids, 15,16-epoxy-8*α*-(benzoyloxy)methyl-2*α*-hydroxycelroda-3,13(16),14-trien-18-oic acid (**10**) and 15,16-epoxy-8*α*-(benzoyloxy)methyl-2-oxocelroda-3,13(16),14-trien-18-oic acid (**11**) isolated from the leaves and stems of *Dodonaca polyandra*, showed maximum inhibition of inflammation (70‒76%) at the dose of 0.10 and 0.39 mg/ear, respectively [70]. Hautriwaic acid (**9**), isolated from *Dodonaca viscosa* leaves, relieved ear edema in TPA-induced mice at the dose of 0.25, 0.5, and 1.0 mg/ear (60.2, 70.2 and 87.1% inhibition, respectively) [71]. Tehuanins G (**11**), isolated from the aerial parts of *Salvia herbacea*, exhibited the comparable effect as that of indomethacin in TPA-induced mice [72]. Tillifodiolide (**40**), isolated from *Salvia tiliifolia*, dose-dependently decreased ear edema in TPA-induced ear edema [73].

Carrageenan is a phlegmatic agent for rat and mice paw edema [74]. Carrageenan-induced paw edema has been described as a local and acute inflammatory process [75]. Caseargrewiin F (**64**) and casearin B (**65**) (0.5 mg/kg) showed a reduction of paw edema in the carrageenan-induced rats, compared to that of indomethacin [76]. *Trans*-dehydrocrotonin (**30**), isolated from the barks of *Croton cajucara* (Euphorbiaceae), alleviated carrageenan-induced paw edema in rats [77]. Tillifodiolide (**40**), isolated from *Salvia tiliifolia* Vahl (Lamiaceae), dose-dependently inhibited paw edema in the carrageenan test [73]. Nepetolide (**43**), a tricyclic clerodane-type diterpenoid isolated from *Nepeta suavis*, reduced carrageenan-induced paw edema at the dose of 20 mg/kg [78]. In carrageenan-induced pleurisy, salvinorin A (**24**) suppressed LTB4 production in exudates, and reduced the phlogistic process in lung [79].

In teleocidin induced mouse ear inflammation, cajucarinolide (**31**) and isocajucarinolide (**32**), two clerodane diterpenoids isolated from the cortices of *Croton cajucara* (Euphorbiaceae), inhibited inflammation in mouse ear at the dosage of 5.6 and 3.0 μg/ear [80].

In TPA-induced acute and chronic mouse ear edema models, polyandric acid A (**10**) from the Australian medicinal plant *Dodonaea polyandra*, inhibited IL-1β and IL-6 production, ear thickness and myeloperoxidase (MPO) accumulation [81].

Stomach illnesses are common health problems worldwide [82]. In indomethacin-induced gastric ulcer rats, epoxy clerodane diterpene (5*R*,10*R*)-4*R*,8*R*-dihydroxy-2*S*,3*R*:15,16-diepoxycleroda-13(16),17,12*S*,18,1*S*-dilactone (**39**), isolated from *Tinospora cordifolia*, showed a gastroprotective effect. Epoxy clerodane diterpene reversed indomethacin-induced increases of ulcer index (UI) and MPO activity, downregulation of PGE2, and decreases of IL-4 and IL-10. Pre-administration of the specific COX-1 inhibitor and nonspecific NOS inhibitor totally blocked the ulcer-healing activity of epoxy clerodane diterpene, indicating the involvement of PGE2 and NOS in the ulcer healing activity of epoxy clerodane diterpene [83].

IBD is caused by microbial infiltration or immunocyte attack, which is a general term for ulcerative colitis and Crohn’s disease [84]. The treatment of IBD remains unsolved. By using dextran sulfate sodium (DSS)/azoxymethane (AOM) induced IBD mouse model, 16-hydroxycleeroda-3,13-dien-15,16-olide (**7**), isolated from *Polyalthia longifolia* var. *pendula* Linn. (Annonaceae), was demonstrated to ameliorate the inflammatory symptoms [85].

Dextran-induced edema induces an anaphylactoid reaction, which is widely used as an acute experimental model of inflammation [86]. *Trans*-crotonina showed inhibitory effect at the rate of 31.9% in dextran-induced edema [80]. Histamine is known to increase vascular permeability [87]. Histamine-induced edema is widely used to screen anti-inflammatory drugs [88]. *Trans*-crotonina showed inhibitory effect in histamine-induced edema [80].

Arachidonic acid-derived LTs are highly related to inflammatory diseases such as asthma, allergic rhinitis and cardiovascular disease [89]. Calcium ionophore A23187-stimulated peritoneal macrophages (PM) were used as an in vitro model to evaluate SA-indcued production of LT [90]. SA concentration-dependently inhibited LTB4 biosynthesis in isolated PM, and suppressed myeloperoxidase activity, cell infiltration, LTC4 production, and vascular permeability, in the peritoneal cavity, but not the production of PGE2 [79].

In summary, a total of 65 clerodane diterpenoids were reported with potential anti-inflammatory property. While, their anti-inflammatory activity was only evaluated on either in vitro cellular models or in vivo animal models, no clinical study was carried out till now. In the future, clinical trials must be performed to authenticate the therapeutic effect of clerodane diterpenoids in alleviating inflammatory responses.

## Comparison of drug-likeness of anti-inflammatory clerodane diterpenoids with marketed drugs

SwissADME is a tool provided by the Swiss institute of Bioinformatics [93], which was used to predict molecular descriptors of the above summarized clerodane diterpenoids. To better summarize the drug-likeness properties of all these compounds, the following descriptors were described, inluding molecular weight (MW), number of hydrogen bond acceptors (HBA) and donors (HBD), number of stereogenic centers, and number of rotatable bonds (RB). The fraction of sp3 carbon (Fsp^3^) is defined as the ratio of sp3 hybrid carbon to the total carbon number. The fraction of carbon and aromatic heavy atoms (Far) is the ratio of the number of aromatic heavy atoms to the total number of heavy atoms [94].

The predicted results were shown in Additional file 1: Table S1, which were grouped according to the previously described categories. Absorption, distribution, metabolism, and excretion (ADME) are critical factors in drug development [95], drug similarity and medicinal chemistry friendliness. Additional file 1: Table S2 showed the physicochemical properties, molecular polar surface area (PSA), pharmacokinetics, LogS and iLOGP, as well as bioavailability characteristics of clerodane diterpenoids (Supplementary Material). Especially for LogP [96] and LogS [97], more than one algorithm was used.

**Fig. 3:**
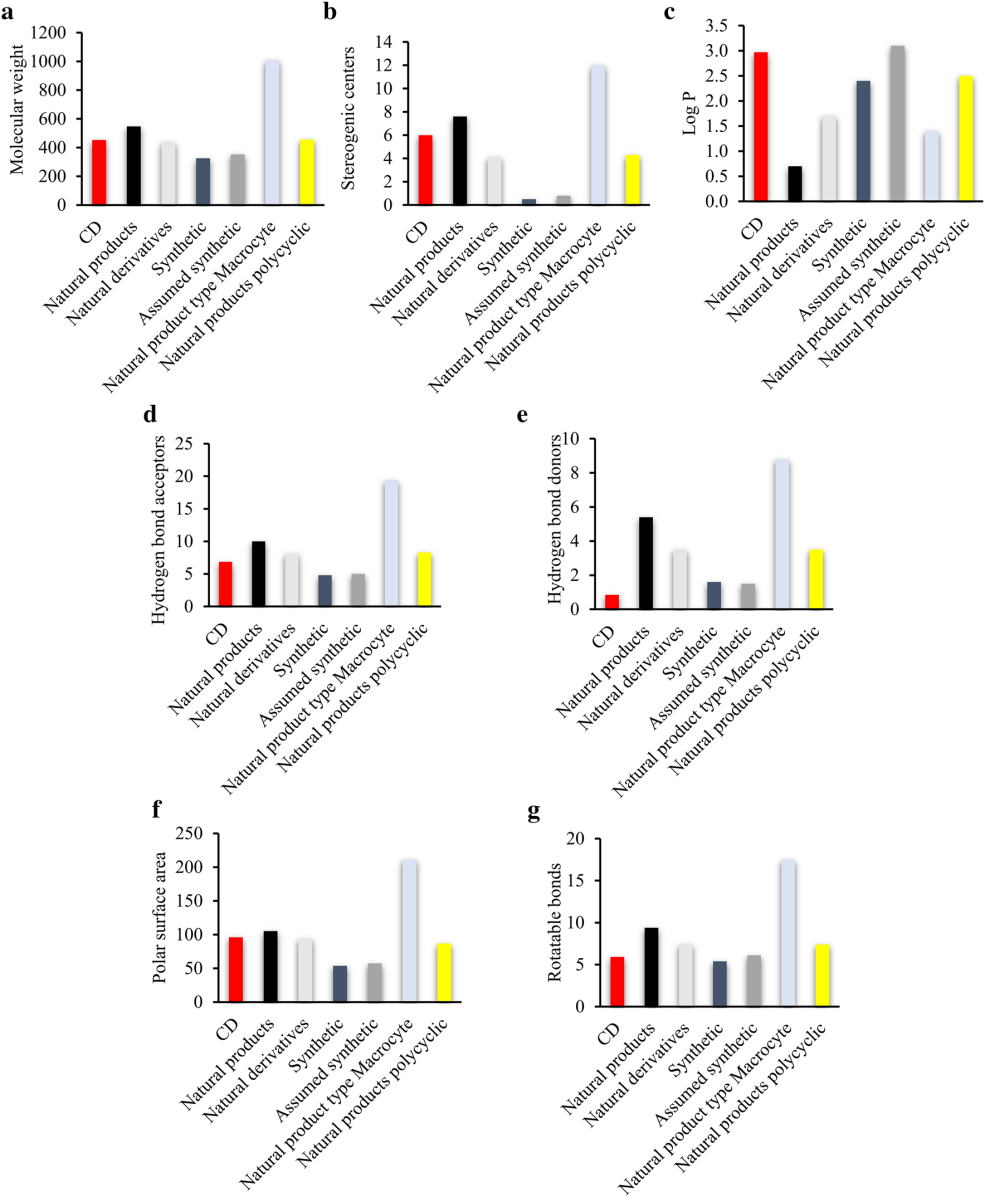
Mean values of MW (**a**), stereogenic centers (**b**), LogP (**c**), HBA (d), HBD (**e**), PSA (**f**), RB (**g**). Anti-inflammatory clerodane diterpenoids (CD) (red), synthetic (dark blue), assumed synthetic (dark grey), natural products (black), natural derivatives (light grey), natural product type macrocyte (light blue), natural products polycyclic (yellow)

To better compare those anti-inflammatory clerodane diterpenoid derivatives with marketed drugs, the MW, HBA, HBD [98], Log P, PSA, RB, and stereogenic centers were evaluated; and the marketed drugs were divided into natural products, natural products derivatives, natural product type macrocycles, polycyclic compounds, synthetic compounds, and assumed synthetic compounds [99, 100] (Fig. 3).

### Size: molecular weight

Lopinski’s rule of five defined the suitable traditional therapeutic agents [101], including less than 500 Da molecular mass, no more than 10 HBA, no more than 5 HBD, and an octanol–water partition coefficient LogP not great than 5. According to the SwissADME results, the mean MW for the anti-inflammatory clerodane diterpenoids was 452.4 Da (Fig. 3a). Most NSAIDs [102], and 61% of clerodane diterpenoids are with the MW less than 500 Da, which match to Lopinski’s rule of five.

### Chirality: number of stereogenic centers

The number of stereocenters has an important impact on the chiral structure and self-assembly of molecular aggregates. The number of stereogenic centers in clerodane diterpenoids was 6, which was comparable with those of natural products, natural products derivatives,natural product type macrocycles, polycyclic compounds, synthetic compounds, and assumed synthetic compounds (Fig. 3b). The natural product type macrocycle have the highest value of the stereogenic center, 12. For the synthesis of chemical compounds, more chiral centers causes more difficulty and higher cost of synthesis [99]. The number of stereogenic centers in the anti-inflammatory clerodane diterpenoid derivatives meets the standards for new drug development.

### Polarity: PSA and HBD/HBA

A major challenge in drug discovery is the prediction of permeability, which, along with solubility, governs the skill of drugs and candidates transport across the gastrointestinal membrane and thus contributes to the overall exposure in systemic circulation and brain penetration. PSA, a descriptor defined as the sum of surfaces of nitrogen or oxygen atoms, plus hydrogen atoms attached to oxygen or nitrogen atoms in a molecule, has been widely used with a discrete success to model permeability and other ADME-related properties [103]. The topological PSA (TPSA) is often implemented in the drug-discovery pipeline [104]. However, the limitation of PSA should be considered. PSA does not take into account the contribution of polarity arising from electronegative atoms in drugs besides nitrogen and oxygen. And the different electronegativity of the atoms of the molecule produces a redistribution of the electron density that in principle involves the entire molecule. PSA is highly correlated with hydrogen bonding (HB. In principle, quantum mechanics calculations could also improve the description of HBA and HBD properties and the polarity of the molecules [105].

**Fig. 4:**
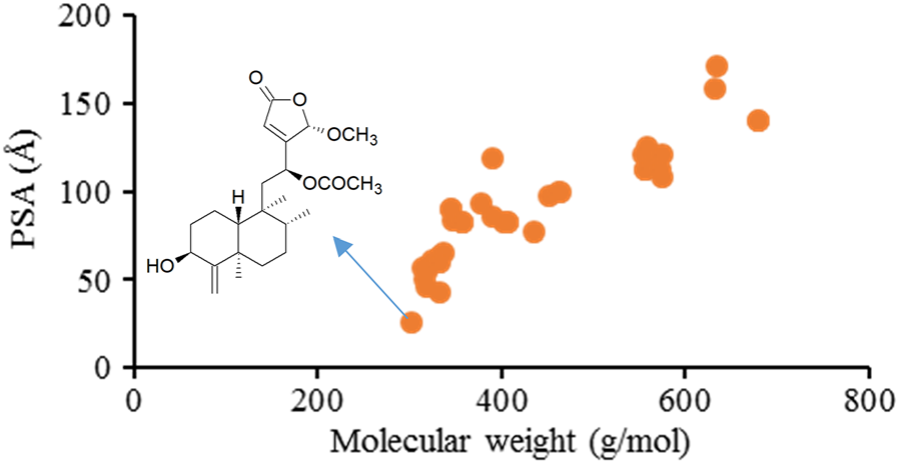
PSA values of the anti-inflammatory clerodane diterpenoid derivatives *vs* molecular weight (MW)

The PSA mean values were 96.1 Å^2^ for clerodane diterpenoids, 86.9 Å^2^ for polycyclic drugs, and 105.3 Å^2^ for natural products. Additionally, the HBA/HBD and PSA values of anti-inflammatory clerodane diterpenoids are positively correlated with their MW (Figs. 3d–f and  4). According to the rule of 5, most of the anti-inflammatory clerodane diterpene derivatives might have good oral absorption.

### Molecular flexibility: rotatable bonds and aromatic character

RBs are the number of bonds that allow free rotation, excluding those adjacent to triple bonds, connect hydrogen or halogen atoms, or in rings containing less than five single bonds [106]. The amide C-N bond is excluded due to its high rotational energy barrier. Reduced molecular flexibility (less RBs), and low PSA or total HB imply good oral bioavailability [107]. The number and percentage of RBs are descriptors for the biological effect of molecules. Compounds with less than 7 RBs exhibited oral bioavailability of more than 20% [108]. The increased RBs number interrupts the permeation rate to reduce oral bioavailability.

The mean numbers of RBs for the summarized clerodane diterpenoids, the polycyclic compounds, natural products, natural product derivatives, and synthetic drugs were 5.92, 7.4, 9.4, 7.4, and 5.4, respectively (Fig. 3g). The less RBs for the clerodane diterpenoids indicated a good permeation rate. The summarized clerodane diterpenoids have a mean Fsp^3^ of 0.64 because they possess an aromatic character.

### Lipophilicity: LogP

LogP is normally determined in the hit to lead stage of drug discovery [109]. Lipophilicity of a drug is the ratio between its concentrations at the equilibrium in 1-octanol and water, commonly described as LogD [110]. The partition coefficient (P) is the specific distribution coefficient (D) at any given pH (typically neutral) [111].

**Fig. 5:**
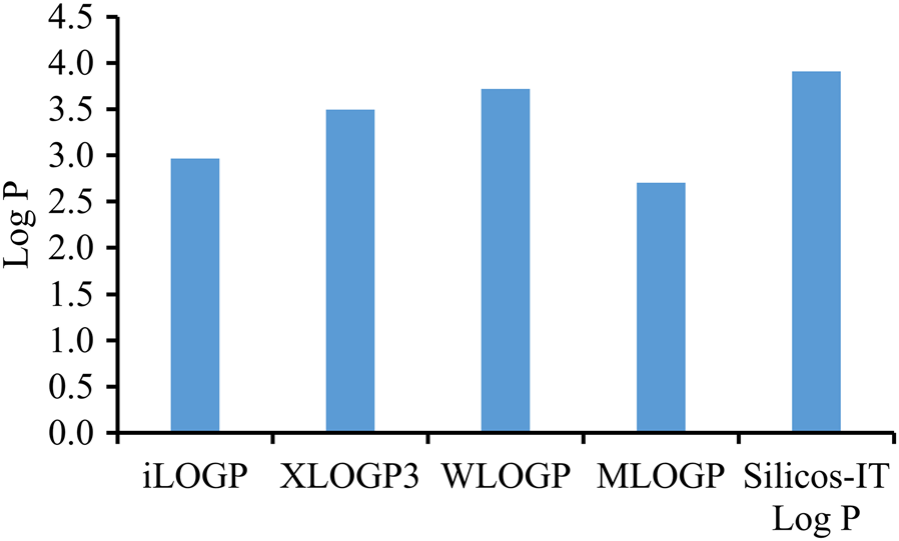
Mean LogP values of the anti-inflammatory clerodane diterpenoids calculated by different methods

The logP values of the anti-inflammatory clerodane diterpenoids are different by using different predicted method on Swiss ADME. Among all logP index, MLOGP is the most discrepant one (Fig. 5). Compare to the synthetic compounds, assumed synthetic compounds, natural products, natural derivatives, natural product macrocycles, and natural products polycyclic, the logP value of the clerodane diterpenoids (3.0) is higher, indicating lower oral bioavailability.

### Solubility: LogS

If the solubility and rate of dissolution are low, an enterally administered drug will mostly be excreted [112]. Solubility of a compound is commonly expressed as logS, where S is the concentration of the compound in a saturated aqueous solution (mol/L). 85% of drugs have logS values between −1 and −5 and virtually none has value below -6 [113].

**Fig. 6:**
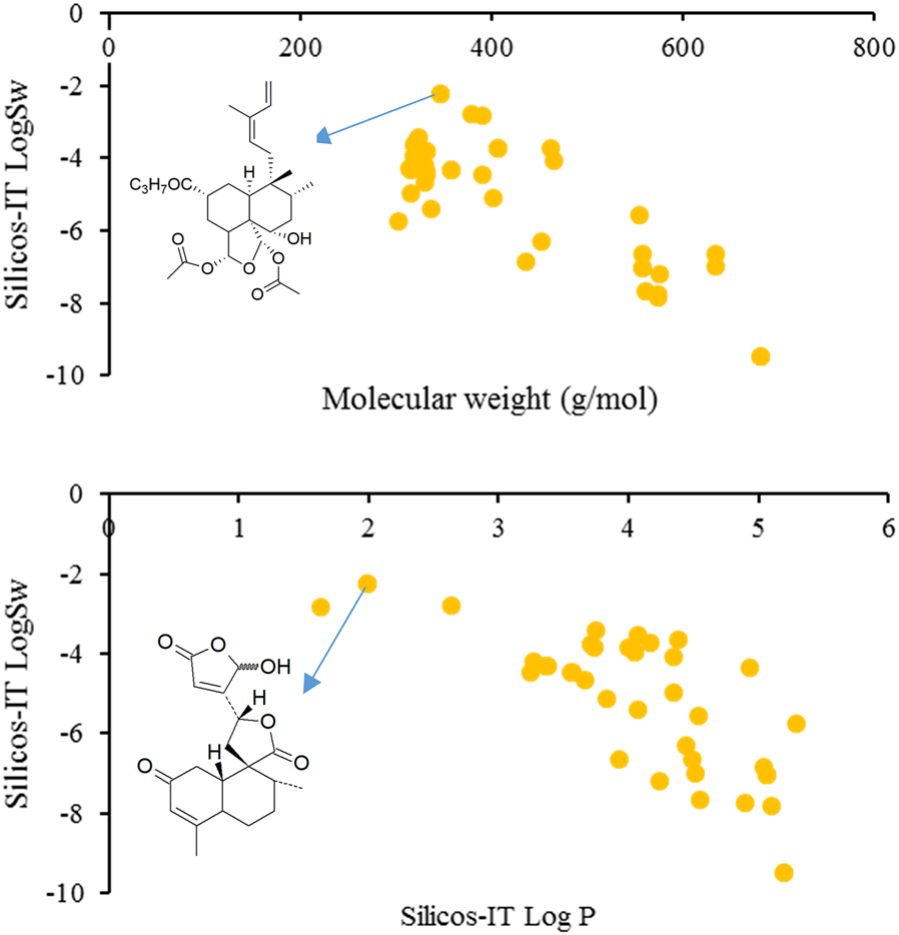
LogS (SILICON-IT) of the anti-inflammatory clerodane diterpenoids vs Molecular weight (top); Log S (SILICON-IT) of the anti-inflammatory clerodane diterpenoids vs LogP (SILICON-IT) (bottom)

The aqueous solubility of clerodane diterpenoids is negatively correlated with MW (Fig. 6). Most of the anti-inflammatory clerodane diterpenoids have poor water solubility. Only a few examples showed the Log S values greater than -4, such as casearin B (**65**) and isocajucarinolide (**32**), which might have great potential to be developed as drugs.

### Compliance of clerodane diterpenoids with the rules of drug-likeness

The “5 Rules” has been widely used as the first filter to eliminate potential drug candidates with poor oral bioavailability [107]. According to different compliance rules, the biophysical and chemical properties and molecular descriptors of the anti-inflammatory clerodane diterpenoids were evaluated. Most of them possess good drug-likeness (green in Additional file 1: Table S3).

### Trends on the PK behavior of clerodane diterpenoids

Besides efficacy and toxicity, poor pharmacokinetics and bioavailability always cause drug development failures. The two pharmacokinetic behaviors, gastrointestinal absorption and brain entry, are critical at different stages of the drug discovery process. BOILED Egg, an accurate predictive model, was developed by calculating the lipophilicity and polarity of small molecules [114]. It is widely used in the filtering of chemical libraries and the evaluation of drug candidates [115].

**Fig. 7:**
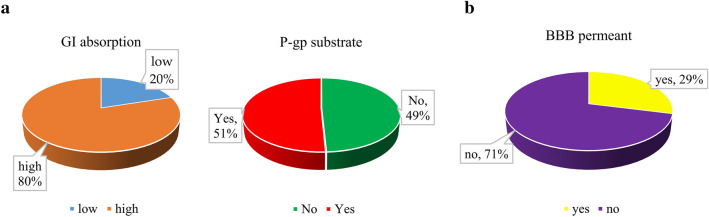
**a** GI absorption for the identified anti-inflammatory clerodane diterpenoids (left pie chart). Anti-inflammatory clerodane diterpenoids with high GI absorption were classified accordingly to its P-gp substrate (right pie chart). (B) BBB permeability of the identified clerodane diterpenoids

Based on the predicted results, about 80% of anti-inflammatory clerodane diterpenoids are more likely to be absorbed in the gastrointestinal (GI), mainly due to their lower MW (Fig. 7a). Among them, 53 clerodane diterpenoids have higher GI absorption, and 34 ones have a high probability as P-gp substrates (Fig. 7a).

The brain capillary endothelium for the blood–brain barrier (BBB) to exclude 100% of large-molecule neurotherapeutics and more than 98% of all small-molecule drug. BBB is the key issue in brain drug development [116]. Most of the anti-inflammatory clerodane diterpenoids have a low probability to cross the BBB (Fig. 7b). Among them, ten compounds are with potential ability to be P-gp substrates (Additional file 1: Table S5).

**Fig. 8:**
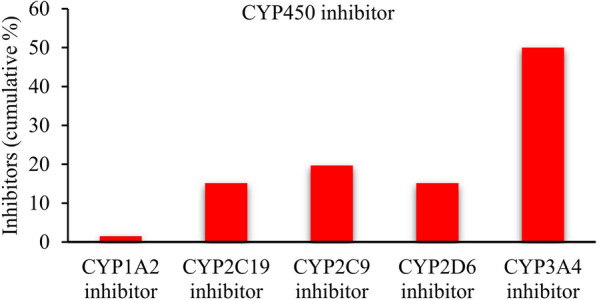
CYP450 enzyme inhibitors of the identified anti-inflammatory clerodane diterpenoid derivatives

The possibility of clerodane diterpenoids as inhibitors of one of the five major isoforms of CYP450 (CYP1A2, CYP2C9, CYP2C19, CYP2D6, and CYP3A4) was predicted by SwissADME (Additional file 1: Table S4) [117, 118]. The anti-inflammatory clerodane diterpenoids are potent CYP450 enzyme inhibitors, especially for the CYP3A4 (Fig. 8).

## Conclusions

Clerodane diterpenoids are secondary metabolites widely distributed in plants, fungi, bacteria, and marine sponges. In this review, 65 anti-inflammatory clerodane diterpenoids were reviewed based on their chemical structures and pharmacological models. The knowledge system of chemistry and bioactivity of clerodane diterpenoids continues to grow rapidly in recent years. Based on the current review, *ent*-clerodane diterpenoids always possess better anti-inflammatory activity than clerodane diterpenoids. And a lactone ring between C-18 and C-19 is essential for the anti-inflammation activity of clerodane diterpenoids. Although lots of evidence suggested the anti-inflammatory property of clerodane diterpenoids, the clinical application of these molecules as anti-inflammatory therapy is still far away. Till now, only evidence from in vitro cellular models or in vivo animal models was available. Clinical trials are necessary to authenticate the therapeutic effect of clerodane diterpenoids in alleviating inflammatory responses. The efficacy of these clerodane diterpenoids is not potent enough; thus, further phytochemical isolation and structural optimization are needed to enhance activity, improve specificity and reduce toxicity. Moreover, virtual docking and/or chemical biology studies should be carried out to identify the potential targets for the anti-inflammatory clerodane diterpenoids. The underlying mechanisms involved in the anti-inflammatory activity should be disclosed by pharmacological investigation.

Online bioinformatics tool SwissADME provides a lot of predicted data of a compound, such as molecular descriptors, the biophysiochemical properties, and the PK parameters. A serious of drug-likeness analyses could be performed to better understand its properties, such as MW, logP, logS, PSA, HBA, HBD, RBs, and number of stereogenic centers. For natural medicine investigation, the combination of bench research and bioinformatics prediction tools could be an effective and economical approach to discover new anti-inflammatory drugs. Regarding to the anti-inflammatory clerodane diterpenoids, future chemical and biological researches will be very bright and challenging, and they have a huge therapeutic application prospect.

## Supplementary information

**Additional file 1: Table S1**.Molecular descriptors of anti-inflammation clerodane diterpenoids. **Table S2**. Biophysiochemical properties of anti-inflammation clerodane diterpenoids.**Table S3**, Violations of drug-likeness rules by the anti-inflammation clerodane diterpenoids. For each compound, the type of violations of each rule was described. **Table S4** .Absorption and Metabolism parameters of anti-inflammation clerodane diterpenoids.

## Data Availability

All available data and material can be accessed.

## Abbreviations

ADME: Absorption, distribution, metabolism, and excretion
BBB: Blood-brain barrier
ECD: Epoxy clerodane diterpene
fMLP: *N*-formylmethionyl-leucyl-phenylalanine
COX: Cyclooxygenase
DSS: Dextran sulfate sodium
GI: Gastrointestinal
HBA, HBD: Hydrogen bond acceptors and donors
HA: Hautriwaic acid
IBD: Inflammatory bowel disease
IL-1β: Interleukin-1β
LTs: Leukotrienes
LTB4: Leukotriene B4
LTC4: Leukotriene C4
LPS: Lipopolysaccharide
MW: Molecular weight
MPO: Myeloperoxidase
NOS: Nitric oxide synthase
NO: Nitric oxide
NOS: Nitric oxide synthase
NSAID and SAID: Nonsteroidal anti-inflammatory drugs (NSAID) and glucocorticoids
PM: Peritoneal macrophages
PGE2: Prostaglandin E2
PSA: Polar surface area
RBs: Rotatable bonds
TNF-α: Tumor necrosis factor-α
TPA: 12-*O*-tetradecanoylphorbol-13-acetate
Th1: T helper type 1
TPSA: Topological polar surface area
UI: Ulcer index
