# Supplementary material for: The drug likeness analysis of anti-inflammatory clerodane diterpenoids
Source: Chin Med. 2020 Dec 9;15:126. doi: 10.1186/s13020-020-00407-w (PMC7727157; doi:10.1186/s13020-020-00407-w)
Supplement: Supplementary file 1 — Additional file 1: Table S1.Molecular descriptors of anti-inflammation clerodane diterpenoids. Table S2. Biophysiochemical properties of anti-inflammation clerodane diterpenoids.Table S3, Violations of drug-likeness rules by the anti-inflammation clerodane diterpenoids. For each compound, the type of violations of each rule was described. Table S4 .Absorption and Metabolism parameters of anti-inflammation clerodane diterpenoids. [file 13020_2020_407_MOESM1_ESM.docx]

**Table S1 Molecular descriptors of marine anti-inflammation clerodane diterpenoids.**

| **No** | **Formula** | **MW** | **Stereogenic Centers** | **#H-bond acceptors** | **#H-bond donors** | **#Rotatable bonds** | **rings** | **Fraction Csp3** |
| --- | --- | --- | --- | --- | --- | --- | --- | --- |
| **1** | C_20_H_30_O_3_ | **318.45** | **5** | **3** | **1** | **5** | **2** | **0.7** |
| **2** | C_20_H_32_O_3_ | **320.47** | **5** | **3** | **2** | **5** | **2** | **0.75** |
| **3** | C_21_H_32_O_3_ | **332.48** | **5** | **3** | **0** | **6** | **2** | **0.71** |
| **4** | C_20_H_36_O_3_ | **324.5** | **7** | **3** | **3** | **4** | **2** | **0.9** |
| **5** | C_20_H_28_O_4_ | **332.43** | **4** | **4** | **1** | **4** | **3** | **0.7** |
| **6** | C_20_H_28_O_4_ | **332.43** | **4** | **4** | **1** | **3** | **3** | **0.7** |
| **7** | C_20_H_30_O_3_ | **318.45** | **4** | **3** | **1** | **3** | **3** | **0.75** |
| **8** | C_20_H_28_O_3_ | **316.43** | **4** | **3** | **1** | **4** | **3** | **0.65** |
| **9** | C_31_H_38_O_9_ | **554.63** | **4** | **9** | **1** | **7** | **3** | **0.65** |
| **10** | C_27_H_32_O_6_ | **452.54** | **5** | **6** | **2** | **8** | **4** | **0.48** |
| **11** | C_27_H_32_O_5_ | **436.54** | **5** | **5** | **1** | **7** | **3** | **0.48** |
| **12** | C_21_H_30_O_6_ | **378.46** | **4** | **6** | **2** | **5** | **3** | **0.71** |
| **13** | C_20_H_28_O_5_ | **348.43** | **4** | **5** | **2** | **4** | **3** | **0.7** |
| **14** | C_32_H_34_N_2_O_7_ | **558.62** | **6** | **9** | **1** | **8** | **5** | **0.41** |
| **15** | C_33_H_35_NO_7_ | **557.63** | **5** | **8** | **1** | **8** | **5** | **0.39** |
| **16** | C_33_H_35_NO_7_ | **557.63** | **5** | **8** | **1** | **8** | **5** | **0.39** |
| **17** | C_35_H_39_NO_10_ | **633.68** | **6** | **11** | **2** | **11** | **5** | **0.46** |
| **18** | C_34_H_38_N_2_O_10_ | **634.67** | **6** | **12** | **2** | **11** | **4** | **0.47** |
| **19** | C_20_H_30_O_2_ | **302.45** | **6** | **2** | **0** | **3** | **4** | **0.8** |
| **20** | C_23_H_34_O_6_ | **406.51** | **7** | **6** | **1** | **6** | **4** | **0.74** |
| **21** | C_23_H_34_O_6_ | **406.51** | **7** | **6** | **1** | **6** | **4** | **0.74** |
| **22** | C_26_H_40_O_7_ | **464.59** | **8** | **7** | **1** | **9** | **4** | **0.81** |
| **23** | C_26_H_38_O_7_ | **462.58** | **7** | **7** | **1** | **8** | **4** | **0.73** |
| **24** | C_23_H_30_O_6_ | **402.48** | **7** | **6** | **0** | **4** | **4** | **0.7** |
| **25** | C_20_H_26_O_4_ | **330.42** | **6** | **4** | **1** | **1** | **4** | **0.65** |
| **26** | C_22_H_30_O_6_ | **390.47** | **7** | **6** | **1** | **3** | **4** | **0.73** |
| **27** | C_20_H_26_O_4_ | **330.42** | **5** | **4** | **1** | **1** | **4** | **0.65** |
| **28** | C_20_H_26_O_4_ | **330.42** | **6** | **4** | **1** | **1** | **4** | **0.65** |
| **29** | C_19_H_24_O_4_ | **316.39** | **6** | **4** | **0** | **1** | **4** | **0.68** |
| **30** | C_19_H_22_O_4_ | **314.38** | **5** | **4** | **0** | **1** | **4** | **0.58** |
| **31** | C_19_H_22_O_6_ | **346.37** | **4** | **6** | **1** | **1** | **4** | **0.63** |
| **32** | C_19_H_22_O_6_ | **346.37** | **5** | **6** | **1** | **1** | **4** | **0.63** |
| **33** | C_32_H_36_N_2_O_7_ | **560.64** | **6** | **9** | **0** | **6** | **4** | **0.53** |
| **34** | C_33_H_37_NO_8_ | **575.65** | **7** | **9** | **1** | **6** | **5** | **0.52** |
| **35** | C_34_H_38_O_8_ | **574.66** | **7** | **8** | **1** | **6** | **4** | **0.5** |
| **36** | C_39_H_40_N_2_O_9_ | **680.74** | **7** | **11** | **0** | **9** | **5** | **0.44** |
| **37** | C_39_H_40_N_2_O_9_ | **680.74** | **7** | **11** | **0** | **9** | **5** | **0.44** |
| **38** | C_34_H_39_NO_7_ | **573.68** | **8** | **8** | **1** | **6** | **5** | **0.53** |
| **39** | C_20_H_22_O_8_ | **390.38** | **2** | **8** | **2** | **1** | **7** | **0.7** |
| **40** | C_20_H_16_O_5_ | **336.34** | **2** | **5** | **0** | **2** | **5** | **0.3** |
| **41** | C_20_H_22_O_6_ | **358.39** | **4** | **6** | **0** | **5** | **4** | **0.55** |
| **42** | C_20_H_22_O_6_ | **358.39** | **4** | **6** | **0** | **5** | **4** | **0.55** |
| **43** | C_20_H_28_O_4_ | **332.43** | **6** | **4** | **1** | **3** | **4** | **0.75** |
| **44** | C_20_H_22_O_6_ | **358.39** | **6** | **6** | **0** | **2** | **5** | **0.6** |
| **45** | C_20_H_22_O_6_ | **358.39** | **6** | **6** | **0** | **2** | **5** | **0.6** |
| **46** | C_21_H_24_O_8_ | **404.41** | **4** | **8** | **1** | **3** | **5** | **0.67** |
| **47** | C_19_H_18_O_9_ | **390.34** | **4** | **9** | **2** | **2** | **5** | **0.53** |
| **48** | C_26_H_36_O_9_ | **492.56** | **7** | **9** | **0** | **10** | **4** | **0.77** |
| **49** | C_29_H_40_O_10_ | **548.62** | **8** | **10** | **1** | **11** | **4** | **0.72** |
| **50** | C_28_H_40_O_9_ | **520.61** | **7** | **9** | **0** | **11** | **4** | **0.79** |
| **51** | C_26_H_36_O_7_ | **460.56** | **7** | **7** | **0** | **9** | **4** | **0.73** |
| **52** | C_33_H_40_O_12_ | **628.66** | **9** | **12** | **0** | **9** | **6** | **0.67** |
| **53** | C_33_H_40_O_12_ | **628.66** | **9** | **12** | **0** | **9** | **6** | **0.67** |
| **54** | C_31_H_38_O_10_ | **570.63** | **8** | **10** | **0** | **7** | **6** | **0.68** |
| **55** | C_22_H_30_O_7_ | **406.47** | **7** | **7** | **2** | **6** | **4** | **0.73** |
| **56** | C_30_H_42_O_10_ | **562.65** | **7** | **10** | **0** | **14** | **3** | **0.7** |
| **57** | C_31_H_38_O_9_ | **554.63** | **8** | **9** | **1** | **7** | **6** | **0.65** |
| **58** | C_33_H_40_O_10_ | **596.66** | **9** | **10** | **0** | **8** | **6** | **0.64** |
| **59** | C_26_H_40_IO_6_ | **575.5** | **6** | **6** | **2** | **9** | **3** | **0.77** |
| **60** | C_30_H_46_IO_9_ | **677.59** | **7** | **9** | **1** | **14** | **3** | **0.8** |
| **61** | C_29_H_46_IO_9_ | **665.57** | **8** | **9** | **3** | **14** | **2** | **0.76** |
| **62** | C_20_H_20_O_6_ | **356.37** | **7** | **6** | **0** | **1** | **5** | **0.6** |
| **63** | C_22_H_24_O_7_ | **400.42** | **6** | **7** | **0** | **3** | **5** | **0.59** |
| **64** | C_29_H_46_O_7_ | **506.67** | **10** | **7** | **1** | **9** | **3** | **0.76** |
| **65** | C_32_H_50_O_10_ | **594.73** | **9** | **10** | **0** | **13** | **3** | **0.75** |

**Table S2 Biophysiochemical properties of marine anti-inflammation clerodane diterpenoids.**

| **No** | **MR** | **TPSA** | **iLOGP** | **XLOGP3** | **WLOGP** | **MLOGP** | **Silicos-IT Log P** | **Consensus Log P** | **Ali Log S** | **ESOL Log S** | **Silicos-IT LogSw** |
| --- | --- | --- | --- | --- | --- | --- | --- | --- | --- | --- | --- |
| **1** | **94.53** | **54.37** | **2.9** | **4.81** | **4.78** | **3.57** | **4.38** | **4.09** | **-5.68** | **-4.51** | **-3.67** |
| **2** | **95.49** | **57.53** | **3.15** | **4.93** | **4.57** | **3.66** | **4.07** | **4.07** | **-5.88** | **-4.6** | **-3.55** |
| **3** | **98.85** | **43.37** | **3.68** | **5.14** | **4.86** | **3.79** | **4.93** | **4.48** | **-5.8** | **-4.74** | **-4.37** |
| **4** | **96.56** | **60.69** | **3.17** | **3.94** | **3.67** | **3.05** | **3.75** | **3.52** | **-4.91** | **-4.07** | **-3.42** |
| **5** | **93.09** | **63.6** | **2.82** | **3.17** | **3.55** | **3.12** | **4** | **3.33** | **-4.18** | **-3.63** | **-3.83** |
| **6** | **93.09** | **63.6** | **2.51** | **3.15** | **3.55** | **3.12** | **3.74** | **3.21** | **-4.16** | **-3.69** | **-3.83** |
| **7** | **92.89** | **46.53** | **3.13** | **4.53** | **4.37** | **4.06** | **4.05** | **4.03** | **-5.23** | **-4.47** | **-3.95** |
| **8** | **92.33** | **50.44** | **3.03** | **5.63** | **5.08** | **3.52** | **4.35** | **4.32** | **-6.45** | **-5.25** | **-4.98** |
| **9** | **143.3** | **120.89** | **3.53** | **3.01** | **3.26** | **2.34** | **4.54** | **3.34** | **-5.21** | **-4.82** | **-5.56** |
| **10** | **124.29** | **96.97** | **3.53** | **5.16** | **4.88** | **3.27** | **4.44** | **4.26** | **-6.94** | **-5.62** | **-6.29** |
| **11** | **123.13** | **76.74** | **3.76** | **6.56** | **5.91** | **4.08** | **5.04** | **5.07** | **-7.97** | **-6.47** | **-6.86** |
| **12** | **100.55** | **93.06** | **2.73** | **3.51** | **3.06** | **2.52** | **2.64** | **2.89** | **-5.15** | **-4.07** | **-2.79** |
| **13** | **94.66** | **83.83** | **2.39** | **3.86** | **3.43** | **3.11** | **2.98** | **3.15** | **-5.32** | **-4.17** | **-2.91** |
| **14** | **149.65** | **124.91** | **2.6** | **3.65** | **4.4** | **2.69** | **4.49** | **3.57** | **-5.96** | **-5.29** | **-6.65** |
| **15** | **151.85** | **112.02** | **3.66** | **4.72** | **5.01** | **3.66** | **5.06** | **4.42** | **-6.8** | **-5.96** | **-7.03** |
| **16** | **151.85** | **112.02** | **3.51** | **4.72** | **5.01** | **3.66** | **5.06** | **4.39** | **-6.8** | **-5.96** | **-7.03** |
| **17** | **164.8** | **158.55** | **3.38** | **4.2** | **4.66** | **2.52** | **4.51** | **3.85** | **-7.24** | **-5.88** | **-7.01** |
| **18** | **162.59** | **171.44** | **2.96** | **3.13** | **4.05** | **1.59** | **3.93** | **3.13** | **-6.4** | **-5.21** | **-6.64** |
| **19** | **90.04** | **25.67** | **3.71** | **5.25** | **5.22** | **3.77** | **5.29** | **4.65** | **-5.54** | **-4.99** | **-5.74** |
| **20** | **109.68** | **82.06** | **3.34** | **3.1** | **3.53** | **2.95** | **3.71** | **3.33** | **-4.49** | **-3.92** | **-3.75** |
| **21** | **109.68** | **82.06** | **3.32** | **3.1** | **3.53** | **2.95** | **3.71** | **3.32** | **-4.49** | **-3.92** | **-3.75** |
| **22** | **124.78** | **99.13** | **4.19** | **4.19** | **3.82** | **3.18** | **4.34** | **3.94** | **-5.98** | **-4.77** | **-4.09** |
| **23** | **124.3** | **99.13** | **4.3** | **3.99** | **3.74** | **3.09** | **4.17** | **3.86** | **-5.77** | **-4.69** | **-3.73** |
| **24** | **106.11** | **82.81** | **2.81** | **3.99** | **3.91** | **2.42** | **3.83** | **3.39** | **-5.43** | **-4.71** | **-5.12** |
| **25** | **90.89** | **59.67** | **2.84** | **2.96** | **3.69** | **2.67** | **3.27** | **3.09** | **-3.88** | **-3.84** | **-4.21** |
| **26** | **102.3** | **85.97** | **3.17** | **2.87** | **3.46** | **2.28** | **3.24** | **3** | **-4.33** | **-4** | **-4.48** |
| **27** | **90.89** | **59.67** | **3.05** | **2.67** | **3.84** | **2.67** | **3.67** | **3.18** | **-3.57** | **-3.66** | **-4.66** |
| **28** | **90.89** | **59.67** | **2.8** | **3.1** | **3.69** | **2.67** | **3.56** | **3.17** | **-4.02** | **-3.93** | **-4.46** |
| **29** | **85.86** | **56.51** | **2.75** | **3.04** | **3.59** | **2.45** | **3.38** | **3.04** | **-3.89** | **-3.81** | **-4.33** |
| **30** | **85.38** | **56.51** | **2.72** | **2.56** | **3.51** | **2.36** | **3.37** | **2.91** | **-3.39** | **-3.5** | **-4.31** |
| **31** | **87.71** | **89.9** | **1.91** | **1.25** | **1.67** | **1.98** | **1.99** | **1.76** | **-2.74** | **-2.71** | **-2.24** |
| **32** | **87.71** | **89.9** | **1.82** | **0.79** | **1.67** | **1.98** | **1.99** | **1.65** | **-2.26** | **-2.42** | **-2.24** |
| **33** | **148.44** | **113.91** | **2.78** | **3.84** | **4.86** | **2.84** | **4.55** | **3.77** | **-5.93** | **-5.56** | **-7.67** |
| **34** | **151.81** | **121.25** | **3.01** | **3.93** | **4.44** | **3.02** | **4.24** | **3.73** | **-6.18** | **-5.7** | **-7.21** |
| **35** | **154.01** | **108.36** | **4.22** | **5.42** | **5.05** | **3.99** | **5.1** | **4.75** | **-7.45** | **-6.63** | **-7.83** |
| **36** | **179.24** | **140.21** | **3.02** | **5.09** | **5.7** | **3.36** | **5.2** | **4.47** | **-7.78** | **-6.94** | **-9.48** |
| **37** | **179.24** | **140.21** | **3.36** | **5.09** | **5.7** | **3.36** | **5.2** | **4.54** | **-7.78** | **-6.94** | **-9.48** |
| **38** | **154.93** | **112.02** | **3.16** | **5.25** | **5.31** | **3.99** | **4.89** | **4.52** | **-7.35** | **-6.52** | **-7.75** |
| **39** | **90.74** | **118.73** | **1.67** | **0.56** | **0.53** | **0.26** | **1.63** | **0.93** | **-2.63** | **-2.68** | **-2.84** |
| **40** | **87.88** | **65.74** | **2.47** | **3** | **2.8** | **2.6** | **4.07** | **2.99** | **-4.04** | **-4.01** | **-5.42** |
| **41** | **91.69** | **82.81** | **2.9** | **1.97** | **3.07** | **1.68** | **3.36** | **2.6** | **-3.33** | **-3.12** | **-4.33** |
| **42** | **91.69** | **82.81** | **2.87** | **1.97** | **3.07** | **1.68** | **3.36** | **2.59** | **-3.33** | **-3.12** | **-4.33** |
| **43** | **91.36** | **59.67** | **2.82** | **3.84** | **3.58** | **2.76** | **3.57** | **3.31** | **-4.79** | **-4.28** | **-4.48** |
| **44** | **90.24** | **74.97** | **2.75** | **1.37** | **2.62** | **1.76** | **2.72** | **2.24** | **-2.55** | **-2.94** | **-4.02** |
| **45** | **90.24** | **74.97** | **2.48** | **1.37** | **2.62** | **1.76** | **2.72** | **2.19** | **-2.55** | **-2.94** | **-4.02** |
| **46** | **96.96** | **112.27** | **2.33** | **1.37** | **1.73** | **1.21** | **2.15** | **1.76** | **-3.33** | **-3.14** | **-3.6** |
| **47** | **88.76** | **128.59** | **1.53** | **0** | **-0.23** | **0.75** | **0.65** | **0.54** | **-2.25** | **-2.13** | **-0.75** |
| **48** | **123.91** | **117.73** | **3.1** | **2.32** | **2.89** | **2.34** | **4.07** | **2.94** | **-4.43** | **-3.7** | **-4.25** |
| **49** | **139.02** | **137.96** | **3.89** | **2.22** | **2.8** | **2.08** | **4.21** | **3.04** | **-4.75** | **-3.91** | **-3.73** |
| **50** | **133.52** | **117.73** | **3.74** | **3.23** | **3.52** | **2.74** | **4.83** | **3.61** | **-5.38** | **-4.38** | **-4.53** |
| **51** | **122.15** | **91.43** | **3.61** | **3.73** | **3.9** | **3.09** | **5.06** | **3.88** | **-5.34** | **-4.45** | **-4.53** |
| **52** | **154.23** | **153.26** | **3.31** | **2.76** | **3.08** | **2.24** | **3.89** | **3.06** | **-5.63** | **-4.98** | **-5.75** |
| **53** | **154.23** | **153.26** | **2.83** | **2.76** | **3.08** | **2.24** | **3.89** | **2.96** | **-5.63** | **-4.98** | **-5.75** |
| **54** | **143.33** | **126.96** | **2.69** | **3.35** | **3.54** | **2.65** | **4.25** | **3.29** | **-5.69** | **-5.13** | **-5.98** |
| **55** | **103.46** | **109.5** | **2.27** | **0.91** | **1.89** | **0.67** | **3.31** | **1.81** | **-2.8** | **-2.66** | **-4.04** |
| **56** | **144.98** | **134.8** | **3.68** | **3.19** | **3.65** | **2.19** | **5.16** | **3.57** | **-5.69** | **-4.41** | **-4.27** |
| **57** | **143.3** | **120.89** | **3.53** | **3.01** | **3.26** | **2.34** | **4.54** | **3.34** | **-5.21** | **-4.82** | **-5.56** |
| **58** | **153.04** | **126.96** | **2.87** | **3.59** | **3.83** | **2.68** | **4.89** | **3.57** | **-5.94** | **-5.38** | **-6.03** |
| **59** | **138.24** | **93.06** | **0** | **4.97** | **4.28** | **3.57** | **5.52** | **3.67** | **-6.66** | **-5.95** | **-5.29** |
| **60** | **159.35** | **125.43** | **0** | **5.03** | **4.48** | **3.5** | **6.01** | **3.8** | **-7.4** | **-6.29** | **-5.88** |
| **61** | **157.22** | **139.59** | **0** | **3.86** | **3.43** | **2.1** | **5.04** | **2.89** | **-6.49** | **-5.47** | **-4.16** |
| **62** | **87.91** | **78.27** | **2.53** | **1.83** | **2.37** | **1.76** | **3.16** | **2.33** | **-3.09** | **-3.28** | **-4.13** |
| **63** | **99.8** | **92.04** | **2.42** | **2.61** | **2.78** | **2.13** | **2.88** | **2.56** | **-4.19** | **-3.9** | **-4.22** |
| **64** | **141.1** | **99.13** | **5.69** | **5.83** | **5.36** | **3.28** | **2.29** | **4.49** | **-7.68** | **-6.06** | **-2.5** |
| **65** | **157.81** | **123.66** | **5.94** | **6.2** | **5.14** | **3.06** | **2.07** | **4.48** | **-8.58** | **-6.58** | **-2.7** |

**Table S3 Violations of drug-likeness rules by the marine anti-inflammation clerodane diterpenoids. For each compound, the type of violations of each rule was described.**

| **No** | **Lipinski** | **Ghose** | **Veber** | **Egan** | **Muegge** |
| --- | --- | --- | --- | --- | --- |
| **1** | **0** | **0** | **0** | **0** | **0** |
| **2** | **0** | **0** | **0** | **0** | **0** |
| **3** | **1** | **1** | **1** | **1** | **1** |
| **4** | **0** | **0** | **0** | **0** | **0** |
| **5** | **0** | **0** | **0** | **0** | **0** |
| **6** | **0** | **0** | **0** | **0** | **0** |
| **7** | **0** | **0** | **0** | **0** | **0** |
| **8** | **1** | **1** | **1** | **1** | **1** |
| **9** | **0** | **0** | **0** | **0** | **0** |
| **10** | **1** | **1** | **1** | **1** | **1** |
| **11** | **1** | **1** | **1** | **1** | **1** |
| **12** | **0** | **0** | **0** | **0** | **0** |
| **13** | **0** | **0** | **0** | **0** | **0** |
| **14** | **0** | **0** | **0** | **0** | **0** |
| **15** | **0** | **0** | **0** | **0** | **0** |
| **16** | **0** | **0** | **0** | **0** | **0** |
| **17** | **3** | **3** | **3** | **3** | **3** |
| **18** | **3** | **3** | **3** | **3** | **3** |
| **19** | **1** | **1** | **1** | **1** | **1** |
| **20** | **0** | **0** | **0** | **0** | **0** |
| **21** | **0** | **0** | **0** | **0** | **0** |
| **22** | **0** | **0** | **0** | **0** | **0** |
| **23** | **0** | **0** | **0** | **0** | **0** |
| **24** | **0** | **0** | **0** | **0** | **0** |
| **25** | **0** | **0** | **0** | **0** | **0** |
| **26** | **0** | **0** | **0** | **0** | **0** |
| **27** | **0** | **0** | **0** | **0** | **0** |
| **28** | **0** | **0** | **0** | **0** | **0** |
| **29** | **0** | **0** | **0** | **0** | **0** |
| **30** | **0** | **0** | **0** | **0** | **0** |
| **31** | **0** | **0** | **0** | **0** | **0** |
| **32** | **0** | **0** | **0** | **0** | **0** |
| **33** | **0** | **0** | **0** | **0** | **0** |
| **34** | **0** | **0** | **0** | **0** | **0** |
| **35** | **1** | **1** | **1** | **1** | **1** |
| **36** | **3** | **3** | **3** | **3** | **3** |
| **37** | **3** | **3** | **3** | **3** | **3** |
| **38** | **1** | **1** | **1** | **1** | **1** |
| **39** | **0** | **0** | **0** | **0** | **0** |
| **40** | **0** | **0** | **0** | **0** | **0** |
| **41** | **0** | **0** | **0** | **0** | **0** |
| **42** | **0** | **0** | **0** | **0** | **0** |
| **43** | **0** | **0** | **0** | **0** | **0** |
| **44** | **0** | **0** | **0** | **0** | **0** |
| **45** | **0** | **0** | **0** | **0** | **0** |
| **46** | **0** | **0** | **0** | **0** | **0** |
| **47** | **0** | **0** | **0** | **0** | **0** |
| **48** | **0** | **0** | **0** | **0** | **0** |
| **49** | **0** | **0** | **0** | **0** | **0** |
| **50** | **0** | **0** | **0** | **0** | **0** |
| **51** | **0** | **0** | **0** | **0** | **0** |
| **52** | **3** | **3** | **3** | **3** | **3** |
| **53** | **3** | **3** | **3** | **3** | **3** |
| **54** | **0** | **0** | **0** | **0** | **0** |
| **55** | **0** | **0** | **0** | **0** | **0** |
| **56** | **0** | **0** | **0** | **0** | **0** |
| **57** | **0** | **0** | **0** | **0** | **0** |
| **58** | **0** | **0** | **0** | **0** | **0** |
| **59** | **0** | **0** | **0** | **0** | **0** |
| **60** | **2** | **2** | **2** | **2** | **2** |
| **61** | **1** | **1** | **1** | **1** | **1** |
| **62** | **0** | **0** | **0** | **0** | **0** |
| **63** | **0** | **0** | **0** | **0** | **0** |
| **64** | **1** | **1** | **1** | **1** | **1** |
| **65** | **1** | **1** | **1** | **1** | **1** |
| **MW – molecular weight, MR - molar refractivity, TNA - total number of atoms, PSA – polar surface area, HBA – hydrogen bond acceptor, HBD – hydrogen bond donor, RB - rotatable bonds, LOG P – considered log P value calculated by XLOGP3.** | | | | | |

**Table S4 Absorption and Metabolism parameters of marine anti-inflammation clerodane diterpenoids.**

| **No** | **GI absorption** | **BBB permeant** | **P-gp substrate** | **CYP1A2 inhibitor** | **CYP2C19 inhibitor** | **CYP2C9 inhibitor** | **CYP2D6 inhibitor** | **CYP3A4 inhibitor** |
| --- | --- | --- | --- | --- | --- | --- | --- | --- |
| **1** | **High** | **Yes** | **No** | **No** | **Yes** | **Yes** | **No** | **Yes** |
| **2** | **High** | **Yes** | **No** | **No** | **Yes** | **Yes** | **No** | **No** |
| **3** | **High** | **Yes** | **No** | **No** | **Yes** | **Yes** | **Yes** | **Yes** |
| **4** | **High** | **Yes** | **No** | **No** | **No** | **No** | **No** | **No** |
| **5** | **High** | **Yes** | **No** | **No** | **No** | **Yes** | **No** | **Yes** |
| **6** | **High** | **Yes** | **Yes** | **No** | **No** | **No** | **No** | **No** |
| **7** | **High** | **Yes** | **No** | **No** | **Yes** | **Yes** | **No** | **No** |
| **8** | **High** | **Yes** | **No** | **No** | **Yes** | **Yes** | **No** | **No** |
| **9** | **High** | **No** | **Yes** | **No** | **No** | **No** | **No** | **Yes** |
| **10** | **High** | **No** | **Yes** | **No** | **No** | **Yes** | **No** | **Yes** |
| **11** | **High** | **No** | **Yes** | **No** | **Yes** | **Yes** | **No** | **Yes** |
| **12** | **High** | **No** | **Yes** | **No** | **No** | **Yes** | **No** | **Yes** |
| **13** | **High** | **No** | **Yes** | **No** | **No** | **Yes** | **No** | **No** |
| **14** | **Low** | **No** | **Yes** | **No** | **No** | **No** | **No** | **Yes** |
| **15** | **High** | **No** | **Yes** | **No** | **No** | **Yes** | **No** | **Yes** |
| **16** | **High** | **No** | **Yes** | **No** | **No** | **Yes** | **No** | **Yes** |
| **17** | **Low** | **No** | **Yes** | **No** | **No** | **No** | **No** | **Yes** |
| **18** | **Low** | **No** | **Yes** | **No** | **No** | **No** | **No** | **Yes** |
| **19** | **High** | **Yes** | **No** | **No** | **No** | **No** | **Yes** | **No** |
| **20** | **High** | **No** | **No** | **No** | **No** | **No** | **No** | **Yes** |
| **21** | **High** | **No** | **No** | **No** | **No** | **No** | **No** | **Yes** |
| **22** | **High** | **No** | **No** | **No** | **No** | **No** | **No** | **Yes** |
| **23** | **High** | **No** | **No** | **No** | **No** | **No** | **No** | **Yes** |
| **24** | **High** | **No** | **No** | **No** | **No** | **No** | **Yes** | **No** |
| **25** | **High** | **Yes** | **Yes** | **No** | **No** | **No** | **No** | **No** |
| **26** | **High** | **No** | **Yes** | **No** | **No** | **No** | **Yes** | **No** |
| **27** | **High** | **Yes** | **Yes** | **No** | **No** | **No** | **No** | **No** |
| **28** | **High** | **Yes** | **Yes** | **No** | **No** | **No** | **No** | **No** |
| **29** | **High** | **Yes** | **No** | **No** | **No** | **No** | **No** | **No** |
| **30** | **High** | **Yes** | **No** | **No** | **No** | **No** | **No** | **No** |
| **31** | **High** | **No** | **Yes** | **No** | **No** | **No** | **No** | **No** |
| **32** | **High** | **No** | **Yes** | **No** | **No** | **No** | **No** | **No** |
| **33** | **High** | **No** | **Yes** | **No** | **No** | **No** | **No** | **Yes** |
| **34** | **High** | **No** | **Yes** | **No** | **No** | **No** | **No** | **Yes** |
| **35** | **High** | **No** | **Yes** | **No** | **No** | **No** | **No** | **Yes** |
| **36** | **Low** | **No** | **Yes** | **No** | **No** | **No** | **Yes** | **No** |
| **37** | **Low** | **No** | **Yes** | **No** | **No** | **No** | **Yes** | **No** |
| **38** | **Low** | **No** | **Yes** | **No** | **No** | **No** | **No** | **Yes** |
| **39** | **High** | **No** | **Yes** | **No** | **No** | **No** | **No** | **No** |
| **40** | **High** | **Yes** | **No** | **Yes** | **Yes** | **Yes** | **Yes** | **Yes** |
| **41** | **High** | **No** | **No** | **No** | **No** | **No** | **No** | **Yes** |
| **42** | **High** | **No** | **No** | **No** | **No** | **No** | **No** | **Yes** |
| **43** | **High** | **Yes** | **No** | **No** | **No** | **No** | **Yes** | **No** |
| **44** | **High** | **Yes** | **No** | **No** | **No** | **No** | **No** | **No** |
| **45** | **High** | **Yes** | **No** | **No** | **No** | **No** | **No** | **No** |
| **46** | **High** | **No** | **Yes** | **No** | **No** | **No** | **No** | **No** |
| **47** | **High** | **No** | **Yes** | **No** | **No** | **No** | **No** | **No** |
| **48** | **High** | **No** | **No** | **No** | **No** | **No** | **Yes** | **No** |
| **49** | **Low** | **No** | **No** | **No** | **No** | **No** | **No** | **Yes** |
| **50** | **High** | **No** | **No** | **No** | **No** | **No** | **No** | **Yes** |
| **51** | **High** | **No** | **No** | **No** | **No** | **No** | **No** | **Yes** |
| **52** | **Low** | **No** | **Yes** | **No** | **No** | **No** | **No** | **Yes** |
| **52** | **Low** | **No** | **Yes** | **No** | **No** | **No** | **No** | **Yes** |
| **54** | **High** | **No** | **Yes** | **No** | **No** | **No** | **No** | **Yes** |
| **55** | **High** | **No** | **Yes** | **No** | **No** | **No** | **No** | **Yes** |
| **56** | **Low** | **No** | **No** | **No** | **Yes** | **No** | **Yes** | **No** |
| **57** | **High** | **No** | **Yes** | **No** | **No** | **No** | **No** | **Yes** |
| **58** | **High** | **No** | **Yes** | **No** | **No** | **No** | **No** | **No** |
| **59** | **High** | **No** | **Yes** | **No** | **No** | **No** | **No** | **No** |
| **60** | **Low** | **No** | **Yes** | **No** | **No** | **No** | **No** | **No** |
| **61** | **Low** | **No** | **Yes** | **No** | **No** | **No** | **No** | **No** |
| **62** | **High** | **No** | **No** | **No** | **No** | **No** | **No** | **No** |
| **63** | **High** | **No** | **No** | **No** | **No** | **No** | **No** | **No** |
| **64** | **High** | **No** | **No** | **No** | **No** | **No** | **No** | **Yes** |
| **65** | **Low** | **No** | **No** | **No** | **Yes** | **No** | **No** | **Yes** |
